# Sequence and phylogenetic analysis of highly pathogenic avian influenza H5N1 viruses isolated during 2006–2008 outbreaks in Pakistan reveals genetic diversity

**DOI:** 10.1186/1743-422X-9-300

**Published:** 2012-12-03

**Authors:** Naila Siddique, Khalid Naeem, Muhammad A Abbas, Zaheer Ahmed, Salman A Malik

**Affiliations:** 1 National Reference Lab for Poultry Diseases, Animal Sciences Institute, National Agricultural Research Centre, Islamabad, 45500, Pakistan; 2 Department of Biochemistry, Quaid-i-Azam University, Islamabad, 45320, Pakistan

## Abstract

**Background:**

Since the first outbreak recorded in northern areas of Pakistan in early 2006, highly pathogenic avian influenza H5N1 viruses were isolated from commercial poultry and wild/domestic birds from different areas of Pakistan up to July 2008. Different isolates of H5N1 were sequenced to explore the genetic diversity of these viruses.

**Results:**

Phylogenetic analysis revealed close clustering and highest sequence identity in all 8 genes to HPAI H5N1 isolates belonging to unified H5 clade 2.2, sub-lineage EMA-3 recovered from Afghanistan during the same time period. Two subgroups within Pakistani H5N1 viruses, from domestic and wild birds, were observed on the basis of their sequence homology and mutations. HPAI motif, preferred receptor specificity for α-(2, 3) linkages, potential N-linked glycosylation sites and an additional glycosylation site at the globular head of HA protein of four Pakistani H5N1 isolates. While, the amino acids associated with sensitivities to various antiviral drugs (Oseltamivir, Zanamivir, Amantadine) were found conserved for the Pakistani H5N1 isolates. Conspicuously, some important mutations observed at critical positions of antigenic sites (S141P, D155S, R162I & P181S) and at receptor binding pocket (A185T, R189K & S217P) of HA-1. A high sequence similarity between Pakistani HP H5N1 and LP H9N2 viruses was also observed. Avian like host specific markers with the exception of E627K in PB2, K356R in PA, V33I in NP, I28V in M2 and L107F in NS2 proteins were also observed.

**Conclusions:**

Various point mutations in different genes of H5 viruses from Pakistan were observed during its circulation in the field. The outbreaks started in Khyber Pakhtoon Khawa (North West) province in 2006 and spread to the Southern regions over a period of time. Though migratory birds may have a role for this continued endemicity of clade 2.2 H5N1 viruses during 2006–2008 in Pakistan, the possibility of their transmission through legal or illegal poultry trade across the borders cannot be ignored.

## Background

Avian influenza (AI) virus infection under certain circumstances poses a serious public health threat and is usually considered a zoonotic infection. In this regard the circulation of highly pathogenic avian influenza (HPAI) virus (H5N1) at a place raises concerns regarding the potential impact of this infection on wild birds, domestic poultry and human health. This subtype has become endemic in poultry in Asian countries since 2003 [1].

Characterization of AIVs as low pathogenic (LP) or high pathogenic (HP) depends on their ability to cause disease in chickens. LP AIV may become HP to poultry through mutations after introduction from wild birds [2]. Until now, only AIV of subtypes H5 and H7 have become HP. There are currently recognized sixteen subtypes of avian influenza viruses (AIVs) on the basis of hemagglutinin (HA) type and nine due to neuraminidases (NAs) [2,3]. Wild waterfowls are the natural reservoirs of all influenza A viruses, however, since late 2002 H5N1 outbreaks in Asia have resulted in mortality among waterfowl in recreational parks, domestic flocks, and wild migratory birds [4].

Indus Flyway (Green Route), which is international migratory bird route number 4, passes through Pakistan and is considered important due to movement of diverse species in large number on this route from Siberia to various destinations in Pakistan. Annually, a variety of migratory bird species come from Europe, Central Asian States and India to Pakistan through this route. Based on regular counts at different Pakistani wetlands, approximately 0.7-1.2 million birds are reported to visit Pakistan through Indus Flyway every year [5].

Avian Influenza was reported in Pakistan for the first time in 1995 when AIV subtype H7N3 was isolated from broiler-breeders during an outbreaks [6]. In 1999, another LP AIV of serotype H9N2 was isolated in breeder flocks from northern parts of the country [7]. Sporadic outbreaks of AIV reported until early 2003 when LP AIV subtypes H7N3 caused repeated outbreaks in commercial poultry; eventually becoming HP few months later with mortality rate as high as 70%. This was controlled by establishment of AI surveillance and diagnosis network along with introduction of aggressive biosecurity at the farms and by undertaking massive vaccination campaign by early 2005 [8]. However, in February 2006 AIV subtype H5N1 was found for the first time in two isolated commercial poultry flocks in Pakistan. The flocks were immediately culled and standard disinfection measures were adopted accordingly [9]. Subsequently, the H5N1 infection spread in the country infecting the commercial poultry, wild birds and backyard poultry resulting in high mortality among infected flocks.

In subsequent years a huge national campaign of strategic vaccination and improved biosecurity at farm level was launched for the control of H5N1 circulation in the country in line with the earlier experience of H7N3. As the LP AIV H9N2 also remained in circulation during H5N1 outbreaks, it was feared that some reassortment in the field could occur, whereby the LP H9N2 may acquire genes from H5N1 to become more pathogenic. Furthermore, as H5N1 circulation was controlled in mid 2008, it still remained to be investigated for any mutation in the circulating strains of H5N1 or introduction of a different clade of H5N1 that may have occurred which affected efficacy of the vaccines used in the control of H5N1.

The present study was designed to carry out sequence and phylogenetic analyses of HPAI H5N1 isolates recovered during 2006–08 from different wild, domestic and commercial poultry populations in Pakistan. This study provided an important insight for understanding the phylogenetic relationship of Pakistani HP H5N1 with the similar subtype isolated from other parts of the world and the possible origin of these viruses.

## Results and discussion

**Figure 1:**
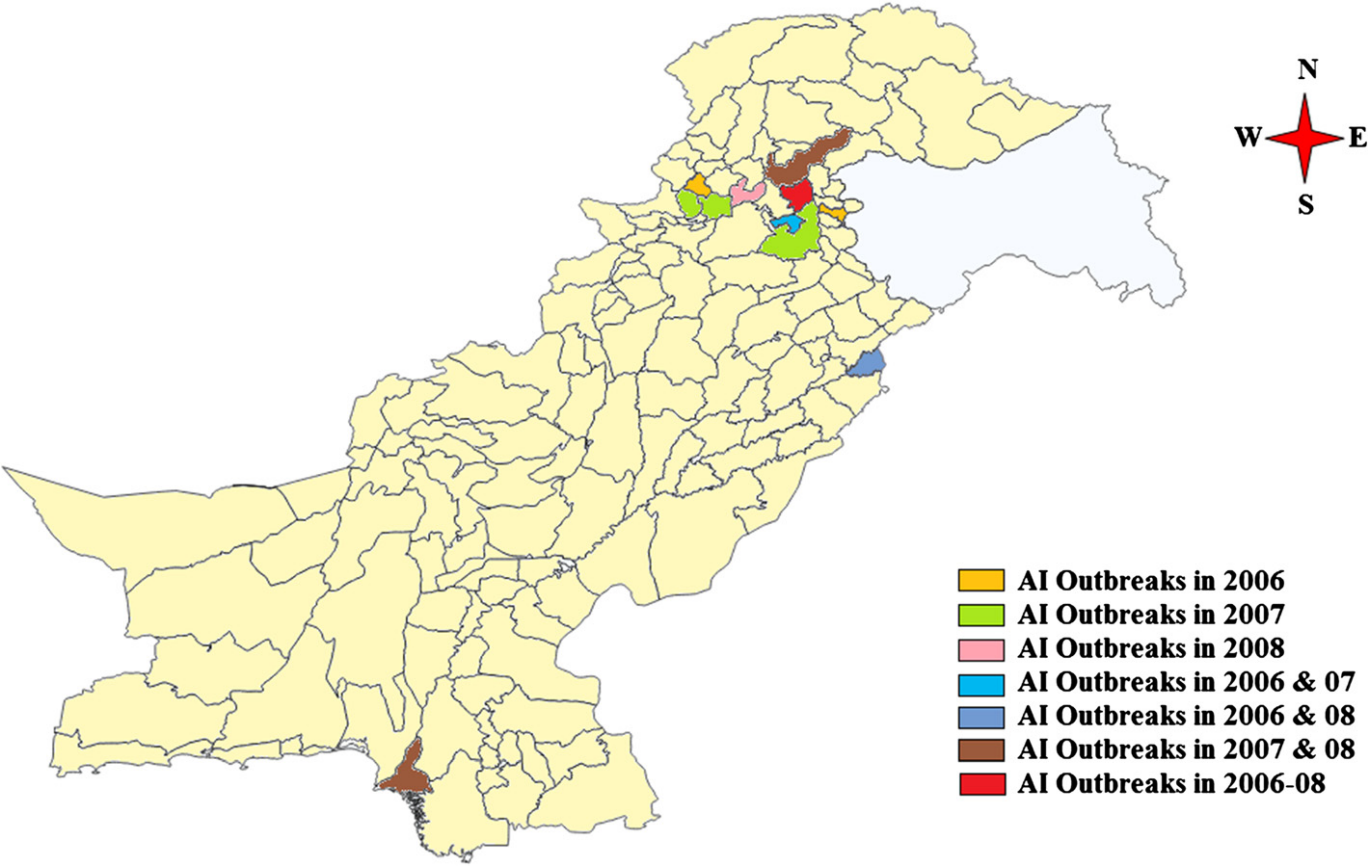
Geographical distribution of HPAI H5N1 outbreaks in Pakistan during 2006–08.

National Reference Lab for Poultry Diseases (NRLPD) at Animal Sciences Institute, National Agricultural Research Center, Islamabad, Pakistan confirmed first case of HPAI H5N1 in a commercial layer flock during February 2006 from northern part of the country. Afterwards, the infection spread to other parts of the country infecting different poultry populations including commercial broilers, breeders, layers, backyard poultry and other domesticated wild birds. Subsequently, outbreaks of antigenically related viruses were reported among variety of wild and migratory birds and backyard poultry along with commercial poultry during the years 2007 and 2008. The only area continuously hit by the HPAI outbreaks during 2006–08 was Abbottabad (Figure 1). The current study was designed to investigate the sequence and phylogenetic relationships of important Pakistani HPAI isolates among each others and with the other important HPAI H5 isolates in the region. The isolates were selected on the basis of difference of the host, area and time of isolation. The details of area, host specie and time of isolation of these selected isolates is provided in Table 1.

**Table 1:** Summarized profile of Pakistani AIV H5N1 isolates used in this study

**S#**	**Viruses nomenclature**	**Summarized profile**						
		**Serotype**	**Date/Year of isolation**	**Place of isolation**	**Host specie**	**Clade**	**Pathogninicity**	**Genes sequenced**
1	A/Ck/Rawalakot/NARC2441/06	H5N1	28-02-2006	Rawalakot, AJK	Chicken	2.2.3	HPAI	Whole Genome
2	A/Ck/Sihala/NARC3303/06	H5N1	14-04-2006	Islamabad, ICT	Breeder	2.2.3	HPAI	Whole Genome
3	A/Ck/Lahore/NARC3320/06	H5N1	18-04-2006	Lahore, Punjab	Broiler	2.2.3	HPAI	Whole Genome
4	A/Gs/Lahore/NARC3321/06	H5N1	18-04-2006	Lahore, Punjab	Goose	2.2.3	HPAI	HA and NA genes
5	A/Pck/Mansehra/NARC-7558/07	H5N1	02-02-2007	Mansehra, KPK	Peacock	2.2.3	HPAI	HA and NA genes
6	A/Gs/Islamabad/NARC-7757/07	H5N1	11-02-2007	Islamabad, ICT	Goose	2.2.3	HPAI	HA and NA genes
7	A/Tk/Islamabad/NARC-7871/07	H5N1	13-02-2007	Islamabad, ICT	Turkey	2.2.3	HPAI	HA and NA genes
8	A/Tk/Islamabad/NARC-7873/07	H5N1	19-02-2007	Islamabad, ICT	Turkey	2.2.3	HPAI	HA and NA genes
9	A/Crow/Peshawar/NARC-7914/07	H5N1	22-02-2007	Peshawar, KPK	Crow	2.2.3	HPAI	HA and NA genes
10	A/Pck/Cahkshahzad/NARC-9238/07	H5N1	21-05-2007	Islamabad, ICT	Peacock	2.2.3	HPAI	HA and NA genes
11	A/Ck/Manshera/NARC-10337/07	H5N1	14-09-2007	Mansehra, KPK	Broiler	2.2.3	HPAI	HA and NA genes
12	A/Ck/Karachi/NARC-520/08	H5N1	08-01-2008	Karachi, Sindh	Layer	2.2.3	HPAI	HA, NA &NS genes
13	A/Ck/Lahore/NARC-610/08	H5N1	08-04-2008	Lahore, Punjab	Layer	2.2.3	HPAI	HA, NA &NS genes
14	A/Ck/Karachi/NARC-612/08	H5N1	15-04-2008	Karachi, Sindh	Layer	2.2.3	HPAI	HA, NA &NS genes
15	A/Crow/Karachi/NARC-11672/08	H5N1	12-02-2008	Karachi, Sindh	Crow	2.2.3	HPAI	HA, NA &NS genes

***ICT***: Islamabad Capital Territory, ***KPK***: Khyber Pakhtoon Khawa, ***AJK***: Azad Jammu & Kashmir, ***Pak***: Pakistan, ***NARC***: National Agricultural Research Center, ***CK***: Chicken, ***Gs***: Goose, ***Pck***: Peacock, ***Tk***: Turkey.

### Sequence and phylogenetic analysis

Detailed sequence and phylogenetic analyses of 8 genes revealed that all the HPAI H5N1 viruses (Abbreviations defined in Table 2) included in this study were related to those circulating in poultry populations of South Asia, East Asia, Middle East and Europe since late 2005. However, highest sequence identities were observed with those viruses originating from neighbouring countries. Phylogenetic analysis revealed close clustering of all the genes with H5N1 isolates from Afghanistan. Other than Afghani isolates, the closest relatives were found to be the isolates from other Eurasian countries. Sequence analysis also revealed a high rate of sequence identity among these Pakistani H5N1 isolates, suggesting a rapid dissemination of the HPAI viruses within the country after single introduction during early phase of these outbreaks in 2006.

**Table 2:** GenBank accession numbers and abbreviations of Pakistani AIV H5N1 isolates used in this study

**Name of isolates**	**Abbreviations used**	**HA**	**NA**	**M**	**NP**	**PA**	**NS**	**PB1**	**PB2**
A/Chicken/Rawalakot/NARC-2441A/06	Ck/Rlkt/2441/06	CY037762	CY037764	CY037765	CY037763	CY037761	CY037766	CY037760	CY037759
A/Chicken/Sihala/NARC-3303/06	Ck/Shla/3303/06	CY037770	CY037772	CY037773	CY037771	CY037769	CY037774	CY037768	CY037767
A/Chicken/Lahore/NARC-3320/06	Ck/Lhr/3320/06	HM208702	HM208704	HM208703	HM208705	HM208707	HM208706	HM208708	HM208709
A/Goose/Lahore-Pakistan/NARC-3321/06	Gs/Lhr/3321/06	CY034733	CY034734	-	-	-	-	-	-
A/Peacockk/Manshera-Pak/NARC-7558/07	Pck/Mnsr/7558/07	EU401796	EU401799	-	-	-	-	-	-
A/Goose/Islamabad/NARC-7757/07	Gs/Isb/7757/07	CY034725	CY034726	-	-	-	-	-	-
A/Turkey/Islamabad-Pak/NARC-7871/07	Tk/Isb/7871/07	EU401794	EU401797	-	-	-	-	-	-
A/Turkey/Islamabad/NARC-7873/07	Tk/Isb/7873/07	CY034727	CY034728	-	-	-	-	-	-
A/Crow/Peshawar/NARC-7914/07	Crow/Psh/7914/07	CY034729	CY034730	-	-	-	-	-	-
A/Peacock/Cahkshahzad/NARC-9238/07	Pck/Cksz/9238/07	CY034731	CY034732	-	-	-	-	-	-
A/Chicken/Manshera/NARC-10337/07	Ck/Mnsr/10337/07	EU401795	EU401798	-	-	-	-	-	-
A/Chicken/Pakistan-Khi/NARC-520/2008	Ck/Khi/520/08	JF827075	JF827076	-	-	-	JF827077	-	-
A/Chicken/Pakistan-Lhr/NARC-610/2008	Ck/Lhr/610/08	JF827078	JF827079	-	-	-	JF827080	-	-
A/Chicken/Pakistan-Khi/NARC-612/2008	Ck/Khi/612/08	JF827081	JF827082	-	-	-	JF827083	-	-
A/Crow/Pakistan-Khi/NARC-11672/2008	Crow/Khi/11672/08	JF827084	JF827085	-	-	-	JF827086	-	-

Not done/Data not available.

**Figure 2:**
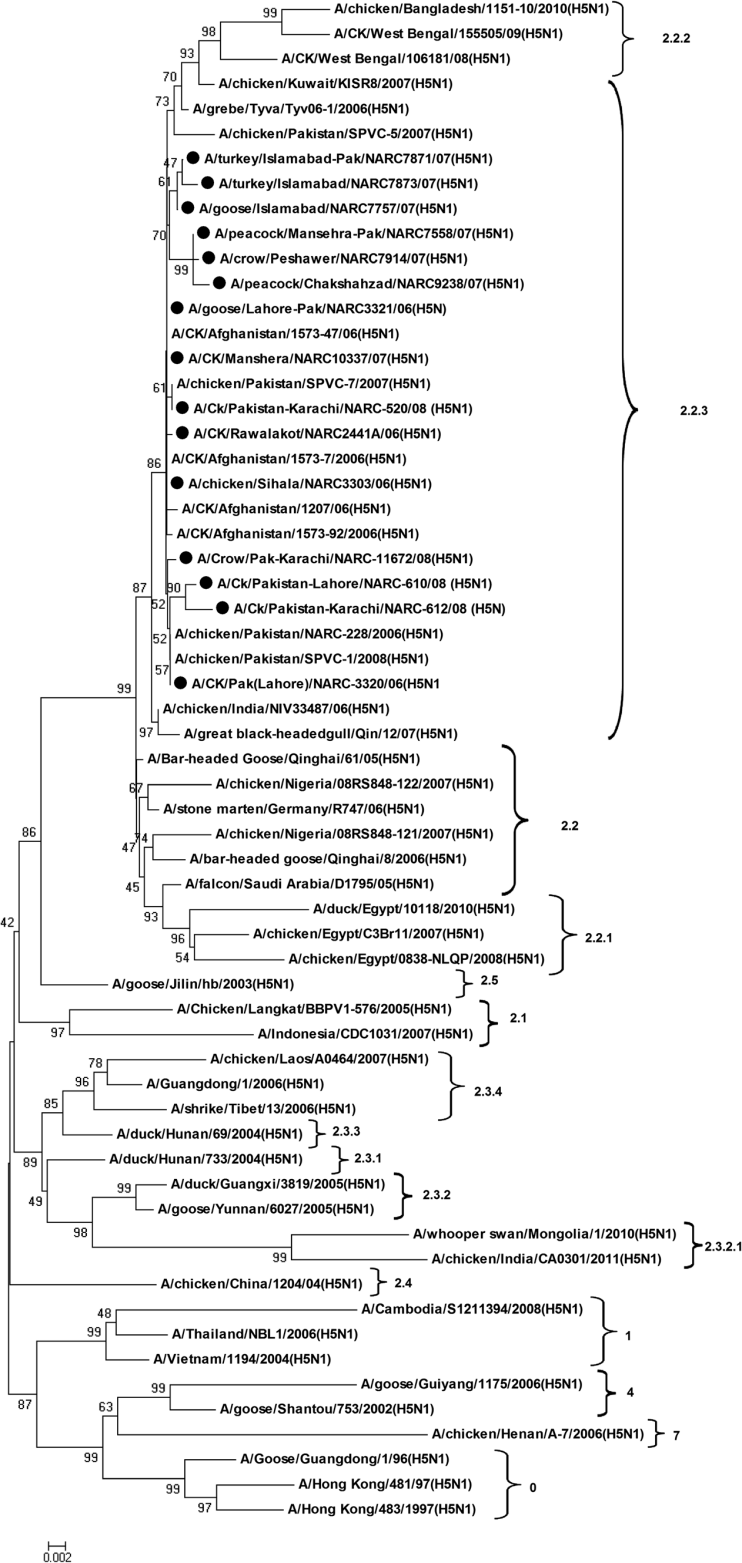
**Evolutionary relationship of HA gene of Pakistani HP H5N1 viruses compared with other representative Eurasian H5N1 viruses.** The nucleotide coding regions tree was generated by neighbour joining method (with Maximum Composite Likelihood) as implemented in MEGA version 4. Numbers at the nodes indicate confidence level of a bootstrap analysis with 1000 replications as a percentage value. Scale bar indicates 0.002 nucleotide substitutions per site. The Pakistani poultry and wild/domestic bird isolates are marked in dark circles. Tree is midpoint rooted.

Phylogenetically HA genes showed tight clustering with Afghani H5N1 isolates (Figure 2) with 99.2-100% sequence homology (Additional file 1). On the other hand one out of 8 of Pakistani wild and domestic birds isolates (Goose/Lahore/3321/06), recovered from the south eastern border near India, formed a cluster with the commercial poultry isolate (Chicken/Mansehra/10337/06) which was recovered from north western Pakistan-Afghanistan border along with two other Afghani isolates (A/Chicken/Afghanistan/1573-47/2006 and A/Chicken/Afghanistan/1573-7/2006), with 100% sequence homology at the nucleotide level. The same isolate also showed close nucleotide resemblance (99.7%) with the Indian H5N1 viruses isolated during 2006 (Additional file 1). These close similarities could be due to the transmission of AIV through migratory birds using green route [5,10] and reflects possibility of a common predecessor originated from wild birds. Moreover, other 6 out of 8 wild and domestic bird isolates, recovered during 2007, formed a separate cluster with two subgroups within the cluster of Pakistani and Afghani H5N1 isolates with sequence similarities of 99.8-99.9% at nucleotide level and 100% homology at amino acid level among them.

**Figure 3:**
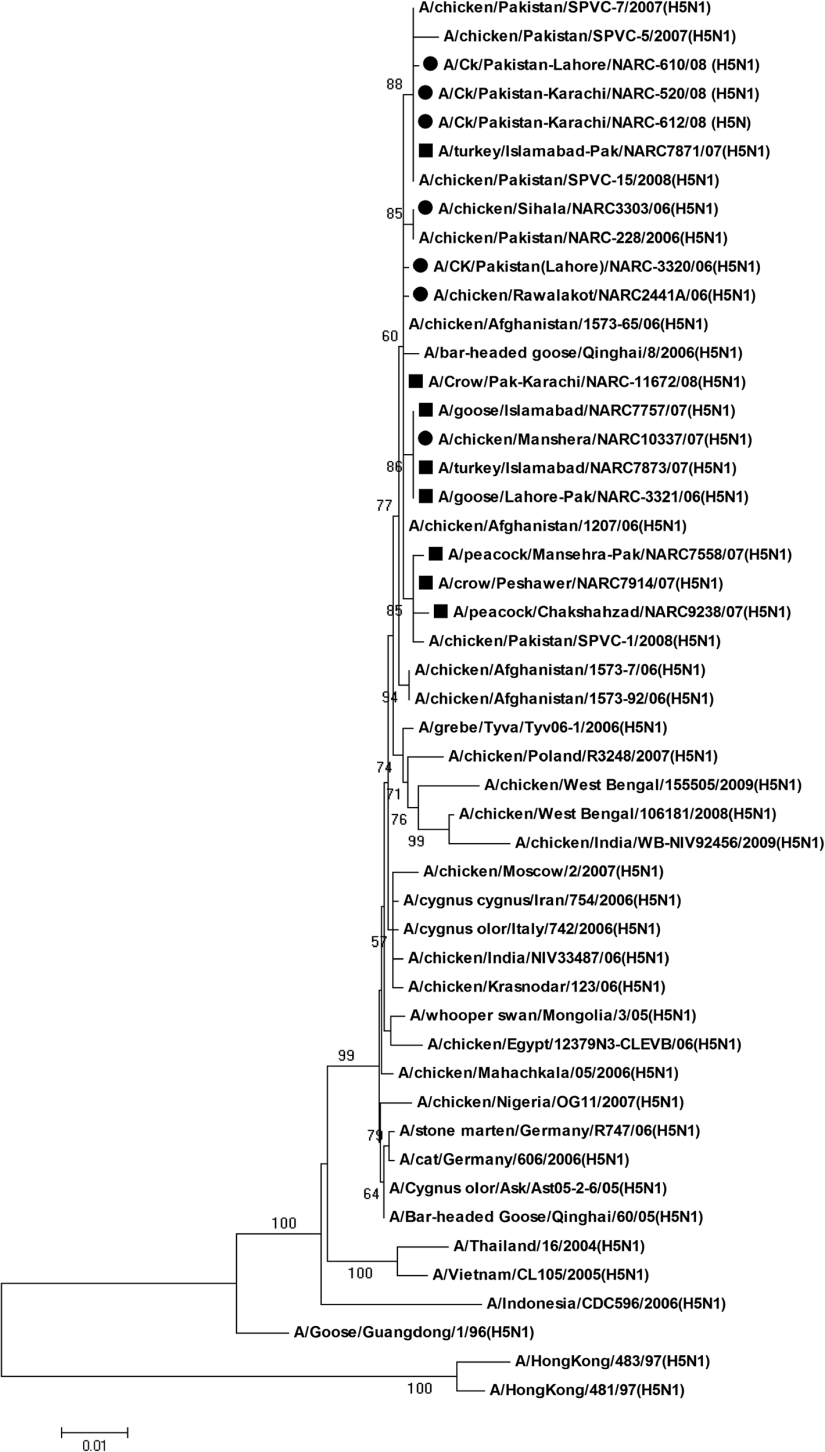
**Evolutionary relationship of NA gene of Pakistani HP H5N1 viruses compared with other representative H5N1 viruses.** The nucleotide coding regions tree was generated by neighbour joining method (with Maximum Composite Likelihood) as implemented in MEGA version 4. Numbers at the nodes indicate confidence level of a bootstrap analysis with 1000 replications as a percentage value. Scale bar indicates 0.01 nucleotide substitutions per site. The Pakistani poultry and wild/domestic bird isolates are marked in dark circles and dark squares respectively. Tree is midpoint rooted.

The NA gene of Pakistani H5N1 AIVs formed a cluster with poultry isolates from Afghanistan, and wild bird isolate from Qinghai showing 99.5-100% sequence identity. However, only one isolate Crow/Karachi/11672/08 clustered with two Afghani isolates (Figure 3) showing 100% sequence homology at the nucleotide level (Additional file 1). The remaining 14 H5N1 isolates showed 99.5- 99.9% sequence identity with the Afghani H5N1 isolates at nucleotide level. Among the NA genes of Pakistani HP H5N1 isolates the sequence homology was 99.4-100% at nucleotide level. The maximum 100% nucleotide sequence identity was observed between three Pakistani isolates from wild and domestic birds (Goose/Islamabd/7757/07, Turkey/Islamabad/7873/07, Goose/Lahore/3321/06) and one isolate from poultry (Chicken/Mansehra/10337/06). While the isolates Chicken/Karachi/520/08, Chicken/Karachi/612/08 and Turkey/Islamabad/7871/07 also showed 100% sequence homology with Chicken/Pakistan/SPVC-15/2008 and Chicken/Pakistan/SPVC-7/2007 at nucleotide level. In addition to these closely related isolates the viruses from Pakistan also showed 97.6-100% sequence identity with those of Asian lineage and 98.3-99.6% with those from European countries. While sequence identity of 94.3-97.3% was also observed with A/Goose/Guangdong/1/96 (H5N1) and other human isolates from Asian countries (Additional file 1). Keeping in view the phylogenetic relation between Pakistani wild & domestic birds and poultry isolates it was suggested that the waves of H5N1 infection occurred during 2006–2008 rapidly spread among Pakistani bird population and no remarkable divergence was observed during three years period. Moreover, all the isolates sequenced in this study showed high sequence homology for HA and NA genes among them during these three waves of H5N1 outbreaks. These close similarities indicate only a single introduction of HA or NA gene in Pakistan [11].

**Figure 4:**
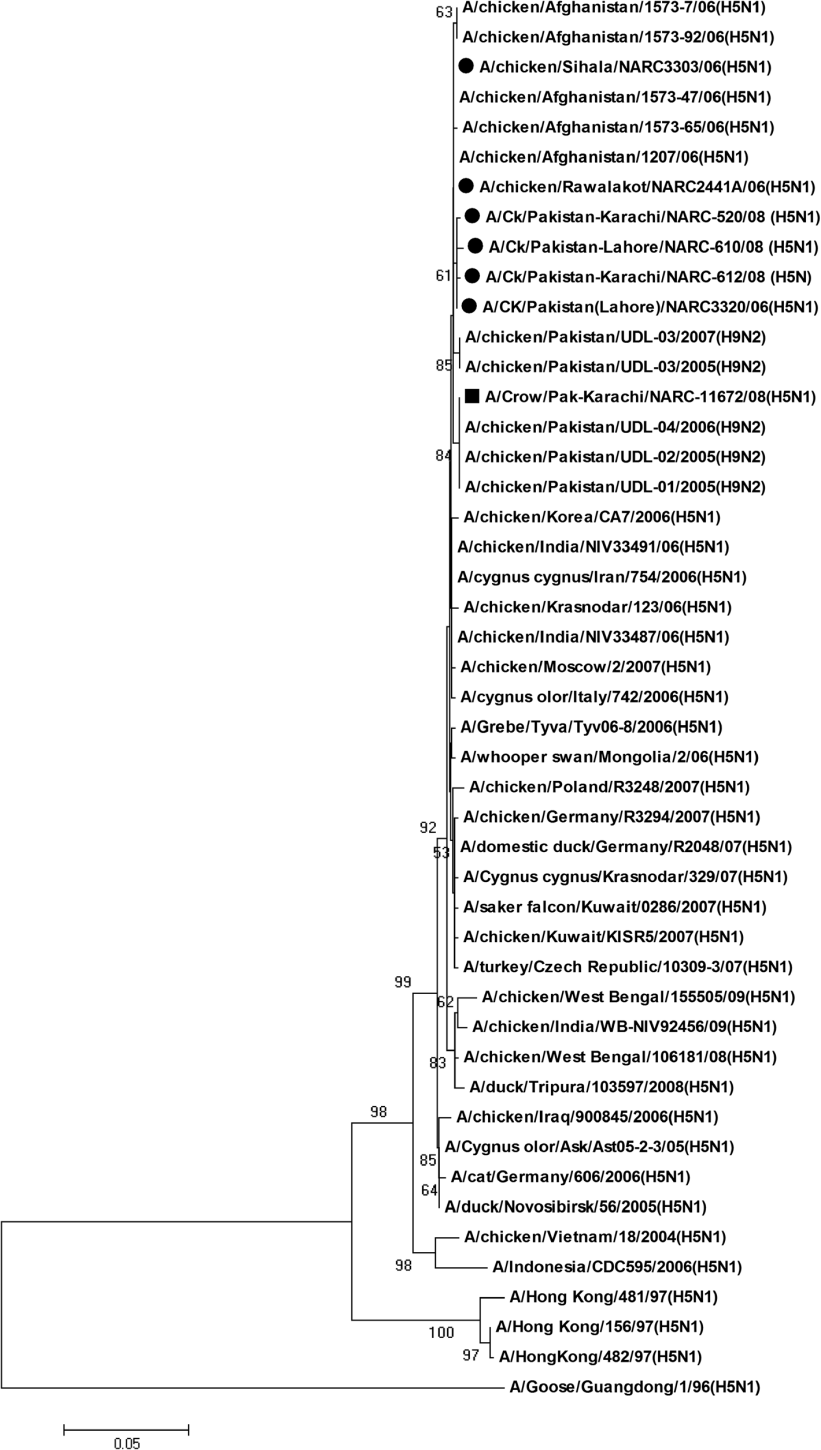
**Evolutionary relationship of NS gene of Pakistani HP H5N1 viruses compared with other representative H5N1 viruses.** The nucleotide coding regions tree was generated by neighbour joining method (with Maximum Composite Likelihood) as implemented in MEGA version 4. Numbers at the nodes indicate confidence level of a bootstrap analysis with 1000 replications as a percentage value. Scale bar indicates 0.05 nucleotide substitutions per site. The Pakistani poultry and wild bird isolates are marked in dark circles and dark squares respectively. Tree is midpoint rooted.

Sequence and phylogenetic analyses revealed that these Pakistani and Afghani HP H5N1 isolates clustered tightly for Matrix, NS, PB1, PB2, NP and PA genes with 99.4-100% sequence homology (Additional file 1, Additional file 2, Additional file 3, Additional file 4, Additional file 5 and Additional file 6. While the NS genes of Pakistani isolates clustered with other H5N1 isolated from the region along with the LPAIs from Pakistan recovered during 2005 (Figure 4). The viruses were found to be 99.4-100% similar with other H5N1olated from Afghanistan and Pakistan for the NS gene (Additional file 1), however, the nucleotide sequence was remarkably similar to other Pakistani LPAI H9N2 [12] as well showing 99.4-100% nucleotides identity (Figure 4, Additional file 1). Overall Matrix, NS, PB1, PB2, NP and PA genes from Pakistani H5N1 viruses were 98.2-99.9% identical for nucleotide sequence with H5N1 isolates from Asian Countries and 98.8-99.8% sequence identity was observed with H5N1 isolates from European countries (Additional file 1).

### Molecular characterization

Pakistani HP H5N1 viruses were classified as Qinghai like viruses and clustered in EMA-3 sub-lineage of defined H5 clade 2.2 according to unified nomenclature [13,14]. Furthermore, Pakistani HP H5N1 viruses isolated during 2006–2007 possessed residues S120, D124, S129, Q138, R140, S141, N154, D155, T159, R162, V174, P181, R189 (H5 numbering), described as important antigenic sites of clade 2.2 HP H5N1 viruses [15]. On the other hand, the viruses isolated during 2008 showed genetic diversity at some of the antigenic sites by acquiring mutations at the amino acid positions S141P, D155S, R162I, P181S and R189K (Table 3). All of 15 Pakistani HP H5N1 isolates had Cleavage motif of QGERRRKKR*GLF with multiple basic amino acids to be described as highly pathogenic [16,17].

**Table 3:** Comparison of antigenically important amino acids in HA genes of Pakistani AIV H5N1 with initially isolated strain during 2006

**Virus names**	**39 E**	**141 S**	**153 K**	**155 D**	**156 A**	**162 R**	**181 P**	**185 A**	**189 R**	**217** **S**	**226** **M**	**252** **N**	**289** **M**	**307** **K**	**376** **D**
Ck/Rawalakot/2441/06	-	-	-	-	-	-	-	-	-	-	-	-	-	-	-
Ck/Sihala/3303/06	G	-	-	-	-	-	-	-	-	-	-	-	-	-	-
Ck/Laore/3320/06	G	-	-	-	T	-	-	-	-	-	-	-	-	-	-
Gs/Lahore/3321/06	G	-	-	-	-	-	-	-	-	-	-	-	-	-	-
Pck/Mansehra/7558/07	G	-	-	-	-	-	-	-	-	-	-	-	-	-	N
Gs/Islamabd/7757/07	G	-	-	-	-	-	-	-	-	-	-	-	K	R	-
Tk/Islamabad/7871/07	G	-	-	-	-	-	-	-	-	-	-	-	K	R	-
Tk/Islamabad/7873/07	G	-	-	-	-	-	-	-	-	-	-	-	K	-	N
Crow/Peshawar/7914/07	G	-	-	-	-	-	-	-	-	-	-	-	-	-	N
Pck/Chakshahzad/9238/07	G	-	-	-	-	-	-	-	-	-	-	-	-	-	N
Ck/Mansehra/10337/07	G	-	-	-	-	-	-	-	-	-	-	-	-	-	-
Ck/Karachi/520/08	G	-	-	-	-	-	-	-	-	P	-	-	-	-	-
Ck/Lahore/610/08	G	P	-	-	T	I	S	-	-	-	-	Y	-	-	-
Ck/Karachi/612/08	G	P	-	G	T	-	S	T	K	-	I	Y	-	-	-
Crow/Karachi/11672/08	G	-	R	-	S	-	-	-	-	-	-	-	-	-	-

*Ck*: Chicken, *Gs*: Goose, *Ty*: Turkey, *Cw*: Crow, *Pk*: Peacock, *Pak*: Pakistan.

- Not done/Data not available.

The conserved residues at positions 222 and 224 (H5 numbering) of HA within the binding pocket determine host range specificity. Pakistani H5N1 isolates not only retained Q222 and G224 (Table 4) but also possessed residues S132, W149, I151, H179, N182, and L190 (H5 numbering) at the receptor binding site, which are known for avian like receptor specificity [18,19]. These results suggested that Pakistani HP H5N1 viruses are well adapted to avian hosts and possessed rare chance to get efficiently transmitted among humans. However, ciliated cells in respiratory epithelium of humans express α-2,3-linked sialic acid receptors, which allow the entry and replication of AI viruses [20]. That might be a cause of reported human cases and death due to H5N1 in Pakistan during 2007 [21,22]. Furthermore, additional multiple sub-grouping was shown by Pakistani H5N1 isolates based upon some point mutations observed in HA gene. In this regard, three of the domestic birds isolates from Islamabad formed subgroup, shared a mutation M289K among them, while mutation K307R was common between the two isolates of the same group. On the other hand four wild and domestic bird isolates from Mansehra, Peshawar, Chakshahzad and Islamabad shared substitution D376N among them (Table 3).

**Table 4:** Various antigenically important amino acid/motifs/deletions observed in different gene segments of Pakistani H5N1 viruses

	**HPAI AA Motif**	**HA 222**	**HA 224**	**NA Deletion** **49-68**	**NA 275**	**MP 26**	**MP 27**	**MP 30**	**MP 31**	**MP 34**	**NS Deletion 80-84**	**NS 149**	**PDZ Binding domain**	**PB2 627**
Ck/Rawalakot/2441/06	PQGERRRKKRGLF	Q	G	YES	H	L	V	A	S	G	YES	A	ESKV	K
Ck/Sihala/3303/06	PQGERRRKKRGLF	Q	G	YES	H	L	V	A	S	G	YES	A	ESKV	K
Ck/Laore/3320/06	PQGERRRKKRGLF	Q	G	YES	H	L	V	A	S	G	YES	A	ESKV	K
Gs/Lahore/3321/06	PQGERRRKKRGLF	Q	G	YES	H	**-**	**-**	**-**	**-**	**-**	**-**	**-**	**-**	**-**
Pck/Mansehra/7558/07	PQGERRRKKRGLF	Q	G	YES	H	**-**	**-**	**-**	**-**	**-**	**-**	**-**	**-**	-
Gs/Islamabd/7757/07	PQGERRRKKRGLF	Q	G	YES	H	**-**	**-**	**-**	**-**	**-**	**-**	**-**	**-**	**-**
Tk/Islamabad/7871/07	PQGERRRKKRGLF	Q	G	YES	H	**-**	**-**	**-**	**-**	**-**	**-**	**-**	**-**	**-**
Tk/Islamabad/7873/07	PQGERRRKKRGLF	Q	G	YES	H	**-**	**-**	**-**	**-**	**-**	**-**	**-**	**-**	**-**
Crow/Peshawar/7914/07	PQGERRRKKRGLF	Q	G	YES	H	**-**	**-**	**-**	**-**	**-**	**-**	**-**	**-**	-
Pck/Chakshahzad/9238/07	PQGERRRKKRGLF	Q	G	YES	H	**-**	**-**	**-**	**-**	**-**	**-**	**-**	**-**	-
Ck/Mansehra/10337/07	PQGERRRKKRGLF	Q	G	YES	H	**-**	**-**	**-**	**-**	**-**	**-**	**-**	**-**	-
Ck/Karachi/520/08	PQGERRRKKRGLF	Q	G	YES	H	**-**	**-**	**-**	**-**	**-**	YES	A	ESKV	-
Ck/Lahore/610/08	PQGERRRKKRGLF	Q	G	YES	H	**-**	**-**	**-**	**-**	**-**	YES	A	ESKV	-
Ck/Karachi/612/08	PQGERRRKKRGLF	Q	G	YES	H	**-**	**-**	**-**	**-**	**-**	YES	A	ESKV	-
Crow/Karachi/11672/08	PQGERRRKKRGLF	Q	G	YES	H	**-**	**-**	**-**	**-**	**-**	YES	A	ESKV	-

*Ck*: Chicken, *Gs*: Goose, *Ty*: Turkey, *Cw*: Crow, *Pk*: Peacock, *Pak*: Pakistan.

Not done/Data not available.

Noticeably, H5N1 viruses isolated during 2008 showed greater genetic diversity and possessed important substitutions in antigenic sites and receptor binding sites of HA1. Specifically the isolate Chicken/Karachi/520/08 possessed mutation S217P (221 in H3 numbering) which has been predicted to be associated with human receptor specificity and suggests a path for H5N1 viruses to gain a foothold in the human population [23]. Whereas, the isolate Chicken/Karachi/612/08 attained two mutations, D155G which is the known antigenic site B [24] and K189R (193 in H3 numbering) described as crucial substitution associated with receptor specificity [23]. In addition, these two isolates were also found for mutation S141P (145 in H3 numbering) which is known as one of the critical residues of site 1 of H5 HA1, an exposed loop that overlaps with antigenic site A of H3 [25] and Ca2 of H1 [26], while mutation of P181S was also observed which is known as important residue of antigenic site in clade 2.2. The isolate Chicken/Lahore/610/08 acquired I162R which is also known as residue in antigenic site of clade 2.2 viruses. Both isolates also attained mutation N252Y while, additionally the isolate A/Chicken/Pak-Karachi/NARC-612/08 possessed two mutations A185T and M226I (Table 3). The isolate Crow/Karachi/11672/08 was found with K153R (157 in H3 numbering), that has been described as a crucial residue of antigenic site 2 of H5 HA1, which corresponds to antigenic site B in H3 serotypes [15].

Pakistani isolates also possessed four potential N-linked glycosylation sites in HA1 at amino acid positions 11, 23, 165, 286 (H5 numbering) along with glycosylation at position 193, which suffers conformational constraints due to the presence of Proline soon after asparagines [23]. The generation of putative glycosylation site as NNT, at amino acid position 156 in human H5N1 isolates has been previously described [27]. Likewise, 156 (H5 numbering) position of HA protein of four Pakistani H5N1 isolates was also mutated (Table 3). The two isolates (Chicken/Laore/3320/06 and Chicken/Lahore/610/08) were mutated from A to T at position 156 but unlike human isolates the glycosylation site generated here was NDT. The isolate Chicken/Karachi/612/08 possessed mutations D155G and A156T and the isolate Crow/Karachi/11672/08 possessed mutation A156S, therefore, the generated glycosylation sites were NGT and NDS, respectively (Table 3). Thus these four isolates possessed an additional glycosylation site which was not present in other Pakistani H5N1 viruses studied so far. The high efficiency of replication could be predicted by the addition of this potential N-Linked glycosylation site at position 156 located on the globular head of the HA in Pakistani H5 viruses [28].

The amino acid residues T183 and N252 in HA protein and R110 in NA protein were also observed in these Pakistani isolates, which are known to be unique for the viruses isolated from migratory birds at Qinghai and Poyang Lakes [29]. The Pakistani H5N1 viruses were observed for having well adaptation characteristics in the poultry [28,30] by the deletion of 20-amino acid in the stalk (position 49–68) of neuraminidase (Table 4). The strains containing Histidine at position 275 of NA (Table 3) were sensitive to the drugs possessing neuraminidase inhibitive effects.

Based upon some unique mutations sub grouping was also observed in the NA genes of Pakistani H5N1 strains. Noticeably, four viruses isolated during 2006–2007 from Mansehra, Lahore and Islamabad formed subgroup also shared two mutations L224M and P340S among them. Additionally, the mutation F74V was common in the group of four viruses isolated during 2007–2008 from Karachi, Lahore and Islamabad. Moreover, the isolates Chicken/Rawalakot/2441/06 and Peacock/Chakshahzad/9238/07 possessed mutations K207R and T383K, respectively, while the isolate Chicken/Sihala/3303/06 attained two mutations S247N and D416G (Table 5).

**Table 5:** Comparison of amino acid mutations observed in NA proteins of Pakistani AIV H5N1 with initially isolated strain during 2006

**Virus name**	**74 F**	**207 R**	**224 L**	**247 S**	**340 P**	**383T**	**416 D**
Ck/Rawalakot/2441/06	-	-	-	-	-	-	-
Ck/Sihala/3303/06	-	K	-	N	-	-	G
Ck/Laore/3320/06	-	K	-	-		-	-
Gs/Lahore/3321/06	-	K	M	-	S	-	-
Pck/Mansehra/7558/07	-	K	-	-	-	-	-
Gs/Islamabd/7757/07	-	K	M	-	S	-	-
Tk/Islamabad/7871/07	V	K	-	-	-	-	-
Tk/Islamabad/7873/07	-	K	M	-	S	-	-
Crow/Peshawar/7914/07	-	K	-	-	-	-	-
Pck/Chakshahzad/9238/07	-	K	-	-	-	K	-
Ck/Mansehra/10337/07	-	K	M	-	S	-	-
Ck/Karachi/520/08	V	K	-	-	-	-	-
Ck/Lahore/610/08	V	K	-	-	-	-	-
Ck/Karachi/612/08	V	K	-	-	-	-	-
Crow/Karachi/11672/08	-	K	-	-	-	-	-

*Ck*: Chicken, *Gs*: Goose, *Ty*: Turkey, *Cw*: Crow, *Pk*: Peacock, *Pak*: Pakistan.

No mutation observed.

The M2 protein possessed residues 26L, 27V, 30A, 31S and 34G, which are known to retained sensitivities to Amantadine [31]. However, no amino acids substitutions associated with resistance to antiviral drugs were detected in the NA and M proteins of Pakistani H5N1 viruses. The substitution E627K was observed in the PB2 gene of Pakistani isolates (Table 4), which has been described as genetic indicator for high pathogenicity in mice and adaptation for efficient replication in humans/mammalian models [32].

These isolates were further characterized by five amino acid deletion (80–84) and also retained amino acid residue A149 in NS1 protein (Table 4). This deletion is known to confer some resistance to the antiviral effects of interferon & tumour necrosis factor, whereas presence of A149 antagonizes the induction of interferon (IFN-α**/**β) in chicken embryo fibroblasts (CEF) [33]. The Pakistani isolates also possessed ESKV amino acid sequence at NS1 Carboxy terminus (Table 4), which is known to be involved in binding to PDZ domains on proteins associated in host cellular signalling pathways [34].

When compared to initially isolated H5N1 virus strain (Chicken/Rawalakot/2441/06) and other closely related Eurasian H5N1 strains, molecular signatures unique to the circulating H5N1 strains isolated in selected areas were also observed in all 8 viral segments sequenced in this study (Tables 3, 4 and 5).

The Pakistani H5N1 viruses also possessed some point mutations in the internal genes. The isolate Chicken/Rawalakot/2441/06 possessed K356R in PA protein, while Chicken/Laore/3320/06 possessed R673M in PA gene. The PB1 gene of three Pakistani H5N1 isolates possessed E739K and Chicken/Sihala/3303/06 possessed substitution M655V in PB1 Gene. The Pakistani H5N1 viruses also shared substitution K221E in the NS gene with Pakistani LP H9N2 viruses and Afghani HP H5N1 viruses. Additionally, the isolate Crow/Karachi/11672/08, which formed a group with three Pakistani LP H9N2 isolates also shared substitution L105I with these LP H9N2 isolate. During 2006–08 H5N1 outbreaks in Pakistan, a number of LP H9N2 viruses were also isolated from the field. The sharing of NS gene among Pakistani LP H9N2 and HP H5N1 viruses has also been described previously [12].

Species specific residues were earlier described in different proteins of Influenza A viruses [35,36]. Pakistani HP H5N1 viruses possessed most of the avian specific residues except E627K in PB2 gene, V33I in NP, I28V in M2 and L107F in NS2 proteins, which are known as human specific residues. Additionally, PA protein of one of the isolates Chicken/Rawalakot/2441/06 possessed mutation K356R, which is typical for human influenza viruses. These human specific mutations in different genes of Pakistani H5N1 strains raise concern to understand the risks posed by H5N1 influenza viruses circulated in birds.

The genetic diversity of Pakistani H5N1 viruses is evident due to some unique mutations observed in all the eight genes, particularly the mutations found at important antigenic and receptor binding sites along with the presence of human like residues. The data is indicative of increased genetic diversity of Pakistani H5N1 isolates from 2006 to 2008 by natural circulation of the virus in the field. Moreover, presence of known molecular signatures associated with pathogenesis, virulence, host specificity, drugs sensitivity, evolution and sharing of unique mutations of Pakistani H5N1 viruses with H5N1 isolates from neighbouring countries indicated that H5N1 virus was circulating continuously in the region and as a consequence has been introduced in Pakistan.

## Conclusions

The data confirmed that clade 2.2 HPAI (H5N1) viruses were responsible for outbreaks in poultry and wild birds recorded in Pakistan during 2006–2008. Notably, the viruses from Pakistan were most closely related to subtype H5N1 isolates from Afghanistan and also showed close relation to H5N1 strains from other neighbouring countries in the region along with those isolated from other Eurasian countries. In this scenario, role of legal and illegal poultry trade across bordering countries and migratory bird movements in the spread of AIV cannot be ignored. The circulation of AIV H5N1 viruses in Pakistan during 2006–2008 not only affected the local economy but also jeopardized animal and human health**.** The genetic diversity observed in Pakistani HP H5N1 viruses during this study demands continued surveillance of poultry and wild birds and constant efforts to monitor the evolution of H5N1 viruses in Pakistan. This would be helpful to minimize the magnitude of any future outbreak and thus limit the risk of human infection. The study might be helpful to increase the knowledge about molecular signatures associated with pathogenesis, virulence, host specificity, drugs sensitivity, genetic diversity and evolution among AIV subtype H5N1. This all may lead to devise better surveillance and control strategies to overcome new introduction of avian influenza viruses in this country and elsewhere.

## Methods

### Isolation and identification of virus

Various organs samples (lungs, trachea, liver, kidney, spleen and brain) along with cloacal and tracheal swabs from dead wild birds/commercial poultry were received from the provincial surveillance units during routine surveillance under the National Program for Control and Prevention of Avian Influenza in the country. The samples were processed and cultured for virus isolation using 9 days old, specific pathogen free (SPF) embryonated chicken eggs for 24–48 hours at 37°C as described previously [37]. Allantoic fluid was harvested 48 hours post inoculation (PI). Standard Hemagglutination (HA) and Hemagglutination inhibition (HI) test were performed using chicken RBC's as described earlier [38,39].

### RNA extraction and genome sequencing

Viral RNA was extracted from the clinical specimens and harvested allantoic fluid, using QIAamp viral mini kit, according to the manufacturer’s instructions (Qiagen, Inc., Valencia CA). Real Time PCR was performed using A/H5 detection kit V-1.0 (Applied Biosystems, Foster City, CA) following the manufacturer's instructions on ABI-7500 Real time PCR systems (Applied Biosystems, Foster City, CA). One step RT-PCR was performed on GenAmp 9700 thermal cycler (Applied Biosystems, Foster City, CA) using Invitrogen Superscript^Tm^ One step RT-PCR with Platinum Taq kit (Invitrogen Inc, USA). The PCR products were separated in an agarose gel (1%) by electrophoresis. Amplicons of the appropriate sizes were subsequently excised from gel and purified, using QIAGEN gel extraction kit (Qiagen, Inc., Valencia CA).

The purified PCR products were directly used for cycle sequencing reactions using BigDye^R^ Terminator v3.1 Cycle Sequencing Kit (Applied Biosystems, Foster City, CA). The products of sequencing reactions were cleaned-up using Performa DTR Ultra 96 Well Plate Kit (Edge BioSystems, USA) and subsequently run on ABI PRISM-3130 Genetic Analyzer (Applied Biosystems, Foster City, CA). The contigs of the nucleotide sequences were generated using SeqScape software version 2.6 (Applied Biosystems, Foster City, CA).

### Phylogenetic analysis

Representative sequences of H5N1 viruses were selected from the GenBank based on sequence identity by BLAST of Pakistani viruses along with other reference strains from various clades of H5N1. Phylogenetic analysis was performed using MEGA version 4 [40]. Multiple nucleotide and amino acid sequence alignments for all eight genes were performed using Clustal W [41]. Phylogenetic trees were generated using neighbour-joining method with the Kimura 2-parameter distance model [42]. To evaluate robustness of nodes, the probabilities of internal branches were estimated by 1000 bootstrap replications. GenBank accession numbers are given in Table 2.

## Competing interests

The authors declare that they have no competing interests.

## Authors’ contributions

NS, KN and ZA conceived this study, NS conducted virus purification, sequencing and phylogenetic analysis. NS, MAA and SAM helped in data interpretation. All the authors contributed in manuscript preparation and its final approval.

## Supplementary Material

Additional file 1 Percentage nucleotides homology of the all genes of Pakistani H5N1 AIVs among them with other closely related AIV strains. Click here for file

Additional file 2 **Evolutionary relationship of M gene of Pakistani HP H5N1 viruses compared with other representative Eurasian H5N1 viruses.** The nucleotide coding regions tree was generated by neighbour joining method (with Maximum Composite Likelihood) as implemented in MEGA version 4. Numbers at the nodes indicate confidence level of a bootstrap analysis with 1000 replications as a percentage value. Scale bar indicates 0.005 nucleotide substitutions per site. The Pakistani isolates are marked in dark circles. Tree is midpoint rooted. Click here for file

Additional file 3 **Evolutionary relationship of NP gene of Pakistani HP H5N1 viruses compared with other representative Eurasian H5N1 viruses.** The nucleotide coding regions tree was generated by neighbour joining method (with Maximum Composite Likelihood) as implemented in MEGA version 4. Numbers at the nodes indicate confidence level of a bootstrap analysis with 1000 replications as a percentage value. Scale bar indicates 0.01 nucleotide substitutions per site. The Pakistani isolates are marked in dark circles. Tree is midpoint rooted. Click here for file

Additional file 4 **Evolutionary relationship of PA gene of Pakistani HP H5N1 viruses compared with other representative Eurasian H5N1 viruses.** The nucleotide coding regions tree was generated by neighbour joining method (with Maximum Composite Likelihood) as implemented in MEGA version 4. Numbers at the nodes indicate confidence level of a bootstrap analysis with 1000 replications as a percentage value. Scale bar indicates 0.01 nucleotide substitutions per site. The Pakistani isolates are marked in dark circles. Tree is midpoint rooted. Click here for file

Additional file 5 **Evolutionary relationship of PB1 gene of Pakistani HP H5N1 viruses compared with other representative Eurasian H5N1 viruses.** The nucleotide coding regions tree was generated by neighbour joining method (with Maximum Composite Likelihood) as implemented in MEGA version 4. Numbers at the nodes indicate confidence level of a bootstrap analysis with 1000 replications as a percentage value. Scale bar indicates 0.005 nucleotide substitutions per site. The Pakistani isolates are marked in dark circles. Tree is midpoint rooted. Click here for file

Additional file 6 **Evolutionary relationship of PB2 gene of Pakistani HP H5N1 viruses compared with other representative Eurasian H5N1 viruses.** The nucleotide coding regions tree was generated by neighbour joining method (with Maximum Composite Likelihood) as implemented in MEGA version 4. Numbers at the nodes indicate confidence level of a bootstrap analysis with 1000 replications as a percentage value. Scale bar indicates 0.02 nucleotide substitutions per site. The Pakistani isolates are marked in dark circles. Tree is midpoint rooted. Click here for file
