# Supplementary material for: Sequence and phylogenetic analysis of highly pathogenic avian influenza H5N1 viruses isolated during 2006–2008 outbreaks in Pakistan reveals genetic diversity
Source: Virol J. 2012 Dec 3;9:300. doi: 10.1186/1743-422X-9-300 (PMC3546873; doi:10.1186/1743-422X-9-300)
Supplement: Additional file 1 — Percentage nucleotides homology of the all genes of Pakistani H5N1 AIVs among them with other closely related AIV strains. [file 1743-422X-9-300-S1.pdf]

Virus Nomenclature	HA Gene	NA Gene	NS Gene	Matrix Gene	NP Gene	PA Gene	PB1 Gene	PB2 Gene
228/SPVC-Pak/06 (H5N1)	99.4-99.9	99.5-100.0	-*	-	-	-	-	-
7/SPVC-Pak/07 (H5N1)	99.3-100.0	99.5-100.0	-	-	-	-	-	-
1/SPVC-Pak/08 (H5N1)	99.5-100.0	99.5-99.8	-	-	-	-	-	-
1573-92/Afg/06 (H5N1)	99.3-99.9	99.4-99.8	99.6-99.9	99.7-100.0	99.7-99.9	99.1-99.8	99.1-99.9	99.1-99.9
1573-7/Afg/06 (H5N1)	99.3-100.0	99.4-99.8	99.6-100	99.6-100.0	99.7-99.9	99.1-99.8	99.1-99.9	99.4-99.9
1207/Afg/06 (H5N1)	99.3-99.8	99.6-100.0	99.8-100	99.7-100.0	99.7-100.0	99.6-99.9	99.1-100	99.1-100.0
10118/Egypt/2010 (H5H1)	97.0-97.6	-	-	-	-	-	-	-
KISR8/Kuwait/07 (H5N1)	98.8-99.4	99.0-99.4	99.1-99.5	99.1-99.5	99.3-99.5	99.4-99.7	99.5-99.6	99.4-99.6
CDC1031/Indonesia/2007 (H5N1)	95.3-95.9	-	-	-	-	-	-	-
33487/India/06 (H5N1)	99.1-99.7	99.2-99.5	99.5-99.8	99.3-99.7	99.5-99.8	99.4-99.7	99.3-99.7	99.6-99.8
1204/China/2004 (H5N1)	96.3-96.9	-	-	-	-	-	-	-
69/Hunan/2004 (H5N1)	96.9-97.4	-	-	-	-	-	-	-
CA7/Korea/06 (H5N1)	99.2-99.6	99.1-99.6	99.2-99.7	99.3-99.6	99.2-99.4	99.3-99.6	99.2-99.4	99.4-99.7
8/Qinghai//06 (H5N1)	98.2-98.9	99.4-99.8	-	-	-	-	-	-
754/Iran/06 (H5N1)	99.0-99.6	99.2-99.6	99.5-99.9	99.3-99.6	99.4-99.8	99.2-99.6	99.5-99.6	99.3-99.6
D1795/Suadi Arabia/05 (H5N1)	98.6-99.2	-	-	-	99.0-99.3	99.1-99.4	99.4-99.5	99.2-99.4
R747/Germany/06 (H5N1)	98.7-99.3	99.0-99.4	-	-	-	-	-	-
02/Mongolia/06 (H5N1)	99.2-99.8	99.2-99.8	-	99.4-99.6	-	99.3-99.7	-	99.6-99.8
C3Br11/Egypt/2007 (H5H1)	98.2-98.9	-	-	-	-	-	-	-
hb/Jilin/2005 (H5N1)	97.7-98.3	-	-	-	-	-	-	-
6-1/Tyva-Tyve/06 (H5N1)	99.2-99.8	99.2-99.6	99.3-99.6	99.6-99.9	99.4-99.6	99.5-99.8	99.6-99.8	99.6-99.8
BBPV1-576/Langkat/2005 (H5N1)	96.3-96.8	-	-	-	-	-	-	-
A0464/Laos/2007 (H5N1)	95.8-96.3	-	-	-	-	-	-	-
106181/India/08 (H5N1)	98.1-98.7	98.5-99.0	99.0-99.3	98.5-99.0	98.8-99.0	99.0-99.2	98.3-99.0	98.4-99.0
733/Hunan/2004 (H5N1)	97.1-97.7	-	-	-	-	-	-	-
156/HongKong/97 (H5N1)	94.6-95.5	86.3-86.8	90.2-90.6	90.2-90.4	90.6-90.8	86.4-86.6	90.0-91.2	83.5-83.8
CA0301/India/2011 (H5N1)	93.1-93.7	-	-	-	-	-	-	-
481/HongKong/97 (H5N1)	94.4-94.9	86.3-86.7	89.6-89.9	90.1-90.4	90.0-90.4	86.2-86.4	90.0-90.2	83.2-83.4
NBL1/Thailand/06 (H5N1)	995.6-96.2	-	-	-	-	-	-	-
1194/Vietnam/2004 (H5N1)	96.3-96.9	-	-	-	-	-	-	-
123/Krasnodar/06 (H5N1)		99.2-99.5	99.3-99.5	99.4-99.6	99.4-99.7	99.4-99.6	99.4-99.6	99.2-99.4
753/Shantou/2002 (H5N1)	95.5-96.0	-	-	-	-	-	-	-
155505/India/09 (H5N1)	97.6-98.1	98.2-98.6	98.3-98.5	98.8-99.0	98.4-98.6	98.4-98.5	98.8-99.0	98.6-98.8
5-2-10/Ask/05 (H5N1)	98.8-99.2	99.2-99.5	-	-	99.4-99.6	-	-	-
02/Novosibirsk/05 (H5N1)	98.9-99.5	-	-	-	-	-	-	-
483/HongKong/97 (H5N1)	94.3-94.8	86.0-86.2	89.4-89.8	90.5-90.8	90.2-90.9	85.8-86.0	90.0-92.2	83.4-83.6
1/shinghai/06 (H5N1)	95.9-99.6	-	-	-	-	-	-	-
216/veitnam/05 (H5N1)	96.2-96.7	-	96.4-96.5	-	-	-	-	-
606/Germany/06 (H5N1)	98.7-99.2	99.0-99.4	99.8-99.0	99.3-99.6	99.2-99.4	99,2-99.4	99.3-99.4	99.0-99.2
3819/Guangxi/2005 (H5N1)	96.3-96.9	-	-	-	-	-	-	-
CDC595/Indonesia/06 (H5N1)		95.9-96.4	-	97.0-97.3	97.5-97.7	96.7.-99.9	97.0-97.2	96.0-96.2
A-7/Henan/2006 (H5N1)	93.0-93.6	-	-	-	-	-	-	-
1/Gaundong/96 (H5N1)	94.9-95.4	96.3-96.7	61.4-61.7	95.7-96.1	91.7-91.9	91.6-91.8	93.0-93.2	93.1-93.4
UDL-01/Pakistan/05 (H9N2)	-	-	99.4-100	-	-	-	-	-
UDL-04/Pakistan/06 (H9N2)	-	-	99.5-100	-	-	-	-	-

*-: Not done/Data not available
