# Supplementary figures and images for: Sequence and phylogenetic analysis of highly pathogenic avian influenza H5N1 viruses isolated during 2006–2008 outbreaks in Pakistan reveals genetic diversity
Source: Virol J. 2012 Dec 3;9:300. doi: 10.1186/1743-422X-9-300 (PMC3546873; doi:10.1186/1743-422X-9-300)

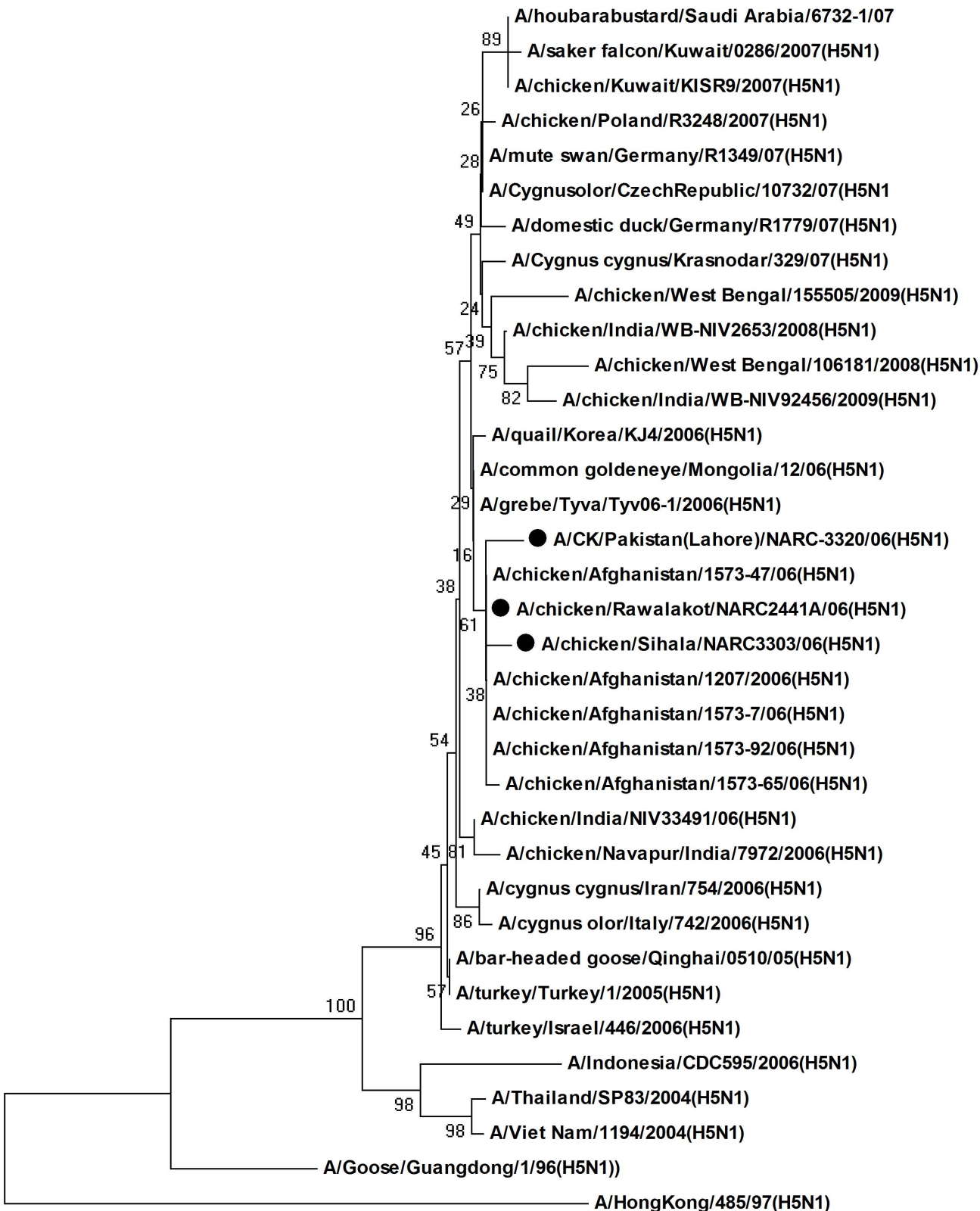

0.005

Supplement: Additional file 2 — Evolutionary relationship of M gene of Pakistani HP H5N1 viruses compared with other representative Eurasian H5N1 viruses. The nucleotide coding regions tree was generated by neighbour joining method (with Maximum Composite Likelihood) as implemented in MEGA version 4. Numbers at the nodes indicate confidence level of a bootstrap analysis with 1000 replications as a percentage value. Scale bar indicates 0.005 nucleotide substitutions per site. The Pakistani isolates are marked in dark circles. Tree is midpoint rooted. [file 1743-422X-9-300-S2.pdf]

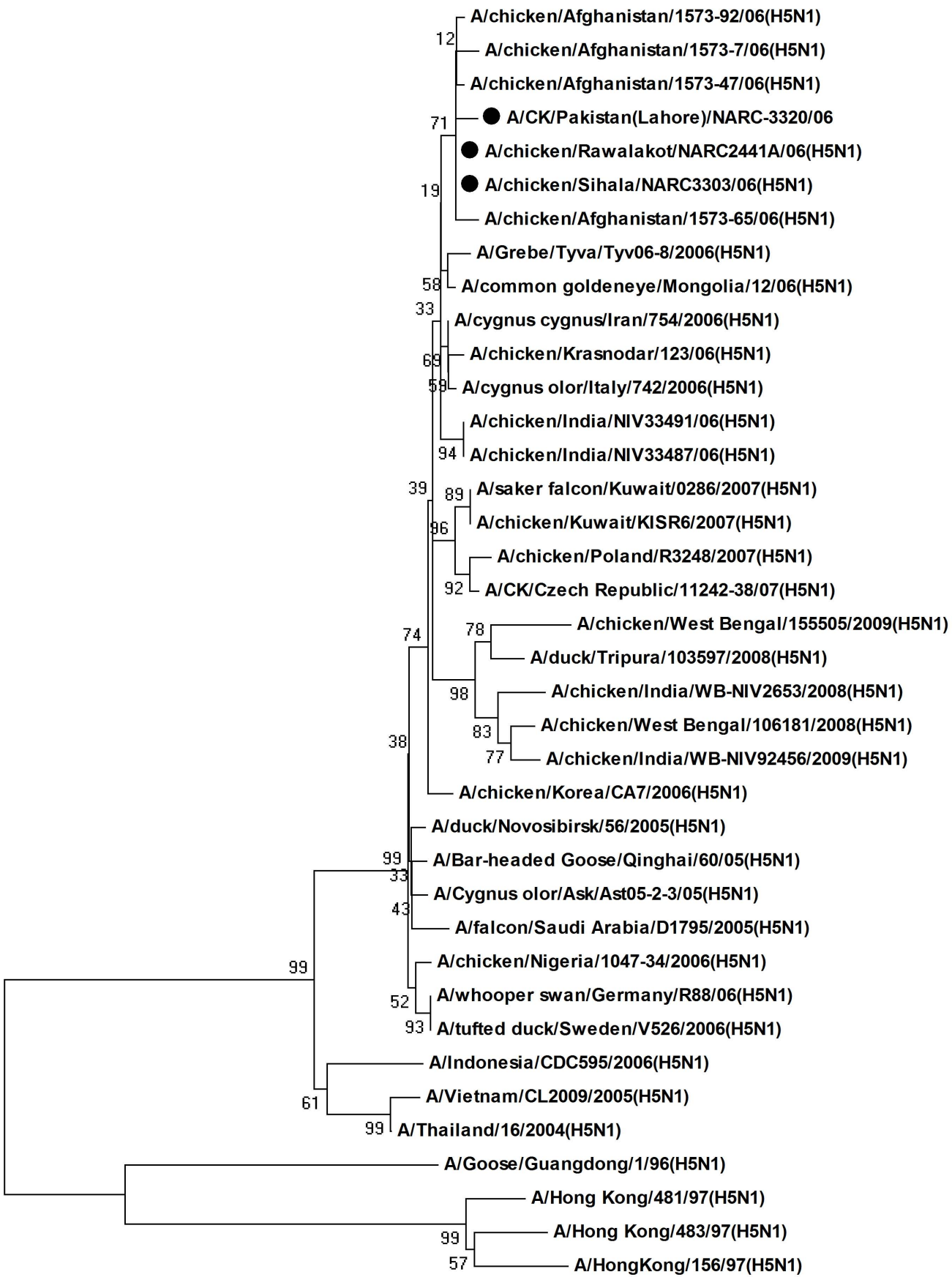

0.01

Supplement: Additional file 3 — Evolutionary relationship of NP gene of Pakistani HP H5N1 viruses compared with other representative Eurasian H5N1 viruses. The nucleotide coding regions tree was generated by neighbour joining method (with Maximum Composite Likelihood) as implemented in MEGA version 4. Numbers at the nodes indicate confidence level of a bootstrap analysis with 1000 replications as a percentage value. Scale bar indicates 0.01 nucleotide substitutions per site. The Pakistani isolates are marked in dark circles. Tree is midpoint rooted. [file 1743-422X-9-300-S3.pdf]

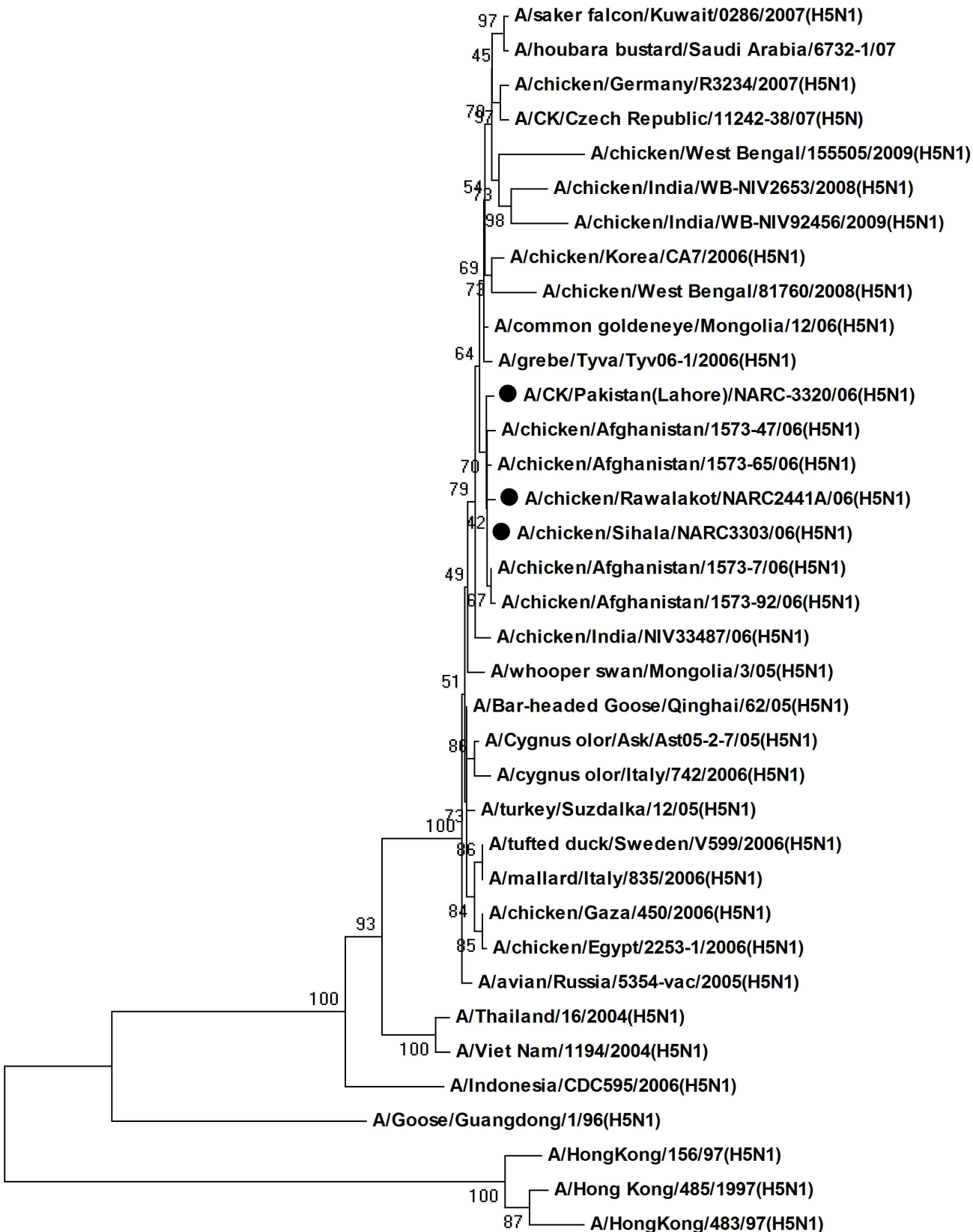

0.01

Supplement: Additional file 4 — Evolutionary relationship of PA gene of Pakistani HP H5N1 viruses compared with other representative Eurasian H5N1 viruses. The nucleotide coding regions tree was generated by neighbour joining method (with Maximum Composite Likelihood) as implemented in MEGA version 4. Numbers at the nodes indicate confidence level of a bootstrap analysis with 1000 replications as a percentage value. Scale bar indicates 0.01 nucleotide substitutions per site. The Pakistani isolates are marked in dark circles. Tree is midpoint rooted. [file 1743-422X-9-300-S4.pdf]

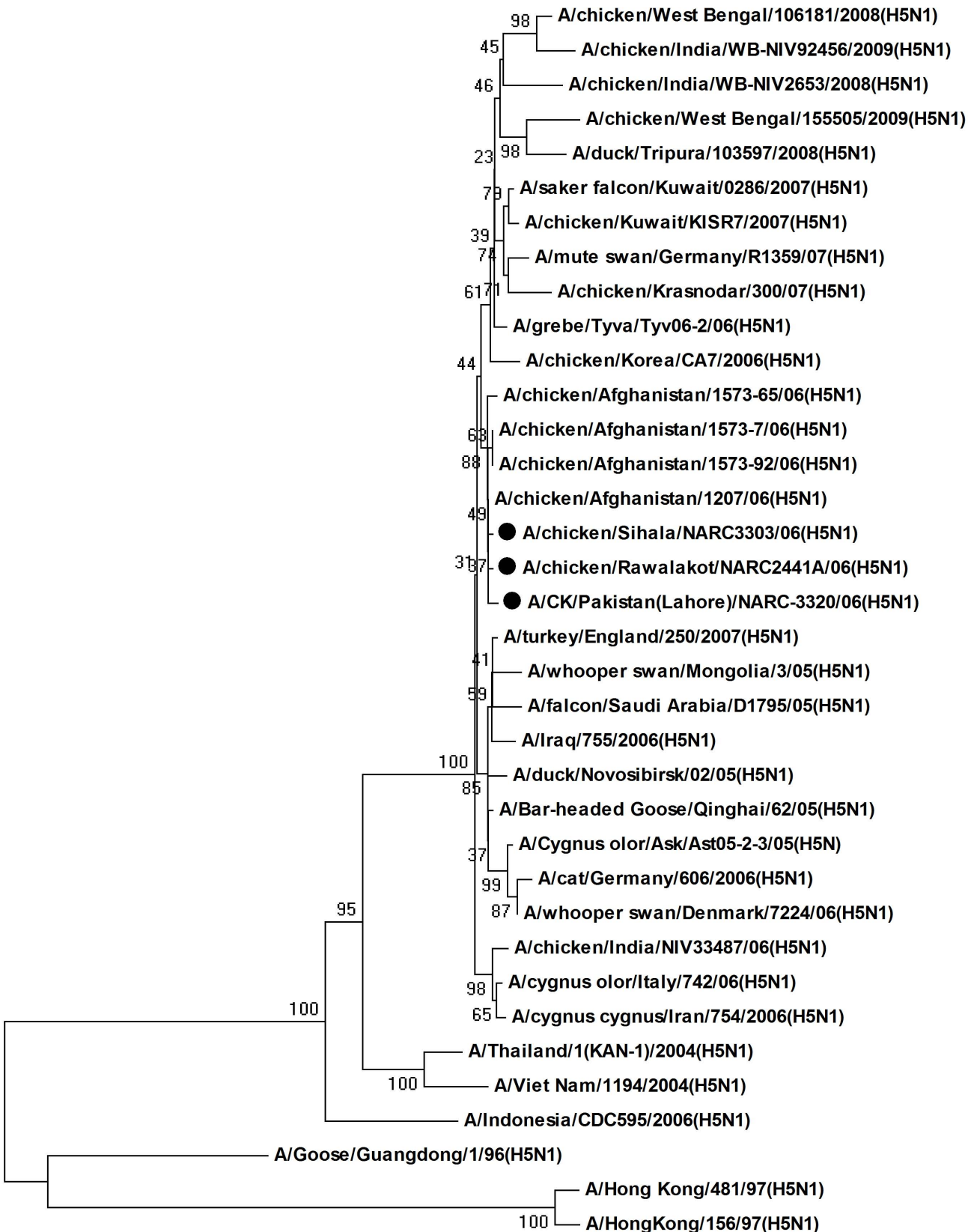

0.005

Supplement: Additional file 5 — Evolutionary relationship of PB1 gene of Pakistani HP H5N1 viruses compared with other representative Eurasian H5N1 viruses. The nucleotide coding regions tree was generated by neighbour joining method (with Maximum Composite Likelihood) as implemented in MEGA version 4. Numbers at the nodes indicate confidence level of a bootstrap analysis with 1000 replications as a percentage value. Scale bar indicates 0.005 nucleotide substitutions per site. The Pakistani isolates are marked in dark circles. Tree is midpoint rooted. [file 1743-422X-9-300-S5.pdf]

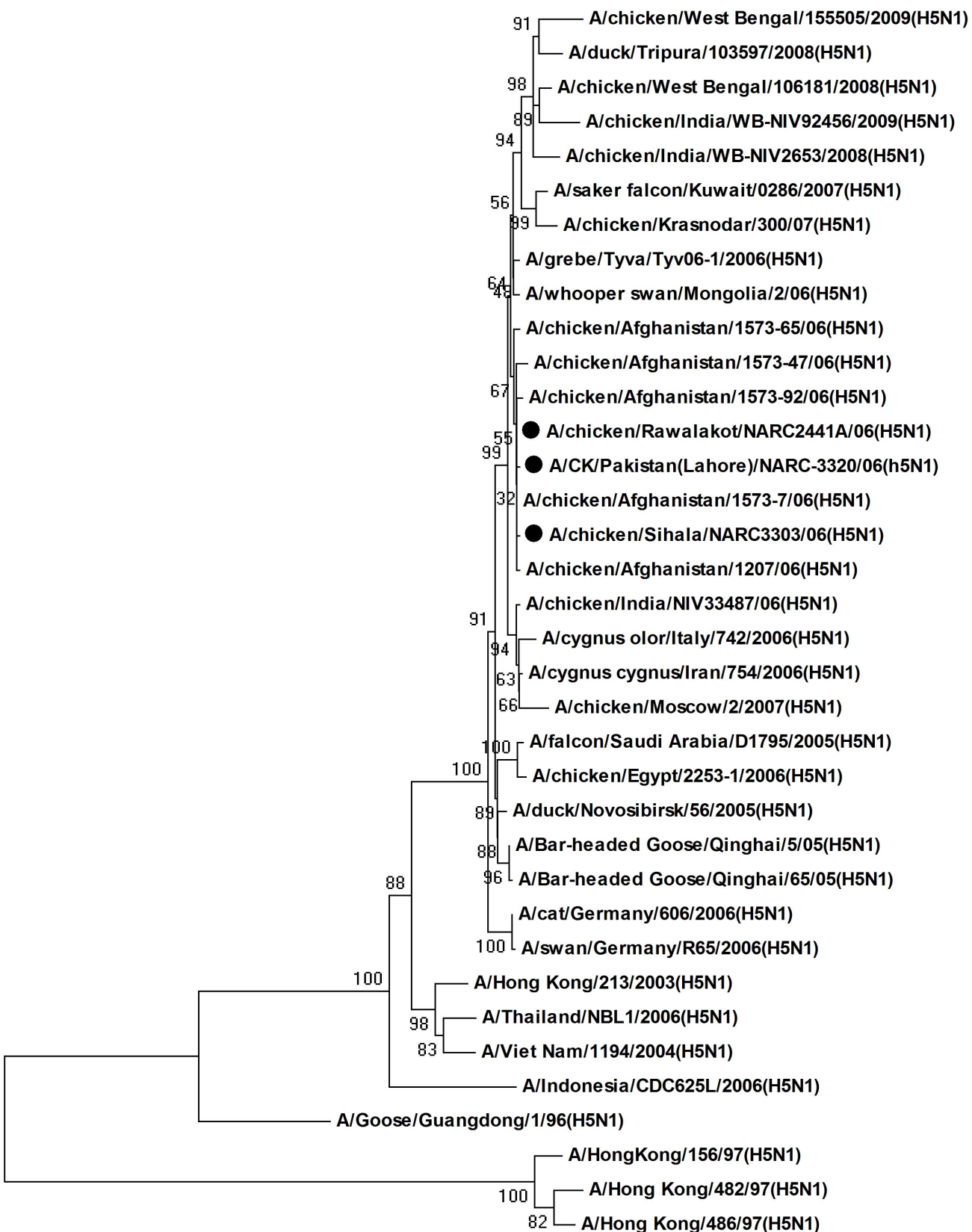

0.02

Supplement: Additional file 6 — Evolutionary relationship of PB2 gene of Pakistani HP H5N1 viruses compared with other representative Eurasian H5N1 viruses. The nucleotide coding regions tree was generated by neighbour joining method (with Maximum Composite Likelihood) as implemented in MEGA version 4. Numbers at the nodes indicate confidence level of a bootstrap analysis with 1000 replications as a percentage value. Scale bar indicates 0.02 nucleotide substitutions per site. The Pakistani isolates are marked in dark circles. Tree is midpoint rooted. [file 1743-422X-9-300-S6.pdf]
